# Prognostic value of quality‐of‐life scores in patients with breast cancer undergoing preoperative chemotherapy

**DOI:** 10.1002/bjs5.50108

**Published:** 2018-11-26

**Authors:** K. Takada, S. Kashiwagi, Y. Fukui, W. Goto, Y. Asano, T. Morisaki, T. Takashima, K. Hirakawa, M. Ohira

**Affiliations:** ^1^ Department of Surgical Oncology Osaka City University Graduate School of Medicine, 1‐4‐3 Asahi‐machi, Abeno‐ku Osaka 545‐8585 Japan

## Abstract

**Background:**

Recently, evaluation of quality of life (QOL) has been recognized as a significant outcome measure in the treatment of several cancers. In this study, the Anti‐Cancer Drugs–Breast (ACD‐B) QOL score was used to assess disease‐specific survival in women with breast cancer undergoing preoperative chemotherapy (POC).

**Methods:**

QOL‐ACD‐B scores were evaluated before and after POC. The cut‐off value of QOL‐ACD‐B contributing to events such as relapse or death was calculated by receiver operating characteristic (ROC) curve analysis.

**Results:**

In 300 women with breast cancer treated with POC, QOL was significantly reduced (*P* < 0·001). A high QOL‐ACD‐B score before POC was an independent factor in the multivariable analysis of overall survival (hazard ratio 0·26, 95 per cent c.i. 0·04 to 0·96).

**Conclusion:**

Evaluation by QOL‐ACD‐B before POC may be useful to predict the prognosis of patients with breast cancer undergoing POC.

## Introduction

Evaluation of quality of life (QOL) has been recognized as an important outcome measure in the treatment of cancer^1^. QOL contributes to ‘health’, defined by the WHO in 1946 as ‘a state of complete physical, mental and social well‐being and not merely the absence of disease or infirmity’^2^. Health‐related QOL is often set as a secondary endpoint in clinical trials. Reporting on health‐related QOL is increasing, with several studies of multiple cancer types indicating that it could affect prognosis^3^, ^4^, ^5^, ^6^.

The QOL scale, Quality of Life Questionnaire for Cancer Patients Treated with Anti‐Cancer Drugs (QOL‐ACD), is a disease‐specific measure supported by the Japanese Ministry of Health and Welfare^7^. QOL‐ACD‐B is an instrument that focuses on patients with breast cancer and the evaluation of treatment^8^.

This study was designed to see whether QOL‐ACD‐B could be used as a prognostic marker in women with locally advanced breast cancer scheduled to receive preoperative chemotherapy (POC). QOL‐ACD‐B scores were measured before and after POC.

## Methods

This study was conducted at the Osaka City University, Graduate School of Medicine, according to the REporting Recommendations for Tumour MARKer Prognostic Studies guidelines (REMARK)^9^. This research was performed in accordance with the provisions of the Declaration of Helsinki (64th World Medical Association General Assembly, Fortaleza, Brazil, October 2013). The protocol was approved by the ethics committee of Osaka City University (approval number 926). Written informed consent was obtained from all patients.

### Patients

Women with locally advanced breast cancer, diagnosed as stage IIA (T1 N1 M0 or T2 N0 M0), IIB (T2 N1 M0 or T3 N0 M0), IIIA (T1–2 N2 M0 or T3 N1–2 M0), IIIB (T4 N0–2 M0) or IIIC (any T N3 M0), and treated with POC between February 2007 and December 2016 at Osaka City University Hospital were included. Patients who started treatment with POC but who could not subsequently undergo surgery were excluded. Initial clinical investigation and restaging after POC included ultrasonography, CT and bone scintigraphy. Breast cancers were classified as subtypes according to the immunohistochemistry expression of oestrogen receptor (ER), progesterone receptor (PgR), human epidermal growth factor receptor (HER) 2 and Ki67, and then categorized as luminal A (ER+ and/or PgR+, HER2−, Ki67‐low), luminal B (ER+ and/or PgR+, HER2+) (ER+ and/or PgR+, HER2–, Ki67‐high), HER2‐enriched breast cancers (HER2BC) (ER–, PgR– and HER2+) and triple‐negative breast cancers (TNBC) (ER–, PgR– and HER2–). Luminal A and luminal B types were considered hormone receptor‐positive breast cancer (HRBC).

### Preoperative chemotherapy

POC consisted of four courses of FEC 100 (500 mg/m^2^ fluorouracil injection (TOWA, Kyoto, Japan), 100 mg/m^2^ epirubicin (Nippon Kayaku, Tokyo, Japan) and 500 mg/m^2^ cyclophosphamide (Endoxan®; Shionogi, Tokyo, Japan)) every 3 weeks, followed by 12 courses of 80 mg/m^2^ paclitaxel (TAXOL® injection; Bristol‐Myers Squibb, New York, USA) administered weekly. In addition, patients with HER2‐positive breast cancer were given trastuzumab (Herceptin®; Chugai, Tokyo, Japan) weekly (2 mg/kg) or every 3 weeks (6 mg/kg) during paclitaxel treatment^10^, ^11^, ^12^.

The antitumour effect of POC was evaluated according to the Response Evaluation Criteria in Solid Tumours (RECIST) within 1 week after completion of POC^13^. Patients with a clinical partial response (cPR) or clinical complete response (cCR) were considered as responders for the objective response rate (ORR). Patients with clinically stable or clinical progressive disease were defined as non‐responders for the ORR.

Women underwent mastectomy or breast‐conserving surgery after POC^14^. The effect of POC was evaluated in resected specimens. A pathological complete response (pCR) was defined as the complete disappearance of invasive components of the lesion, with or without intraductal components, including that in the lymph nodes, according to the National Surgical Adjuvant Breast and Bowel Project B‐18 protocol^15^. All patients who had breast‐conserving surgery received postoperative radiotherapy to the remnant breast tissue. The standard postoperative adjuvant therapy was chosen based on the intrinsic disease subtype.

### QOL‐ACD‐B

QOL‐ACD‐B includes 18 items with four subscales: Physical symptoms and pain (6 items); Satisfaction with treatment and coping with disease (4 items); Side‐effects of treatment (4 items); and Dress, sexual aspect, other (4 items) (*Table* 1). Patients answer questions by checking the number on the scale that best describes their state. Each item is evaluated by scores of 1–5, with 1 being the worst and 5 the best. Scores for the whole QOL questionnaire and each subscale were calculated by subtracting 1 from the mean of the items checked and multiplying by 25, so that the minimum value was 0 and the maximum 100.

**Table 1:** Quality‐of‐Life Questionnaire for Cancer Patients Treated with Anti‐Cancer Drugs–Breast (QOL‐ACD‐B)

Physical symptoms and pain	
1	Did you have pain or numbness in the chest, armpits or arms on the diseased side?
2	Did you have swollen arms on the diseased side?
3	Were you able to raise your arm completely on the diseased side?
4	Were you concerned about the skin symptoms (redness, swelling, hotness, itching, etc.) around the chest on the diseased side?
5	Did you have any pain related to disease or treatment?
6	(Please answer this question only if you had surgery) Were you satisfied with the appearance of your breasts and surgical scar?
Satisfaction with treatment and coping with disease	
7	Were you satisfied with the explanation from your doctor about your medical condition and treatment?
8	Were you satisfied with the hospital facilities and staff other than doctors?
9	Do you feel that you have adequately accepted your disease?
10	Have you tried to face up to the disease positively?
Side‐effects of treatment	
11	Did you have hair loss?
12	Did you feel tired?
13	Did you suffer from hot flushes and sweating of your body and forehead?
14	Did you suffer from changes in taste (abnormalities)?
Dress, sexual aspect, other	
15	Do you find it difficult to wear the clothes you want to wear?
16	Do you feel hesitant about undressing in public, such as at a hot spring?
17	Are you satisfied with your sex life?
18	Are you worried that your family will get the same disease?

In this study, QOL‐ACD‐B was used to evaluate QOL retrospectively. Initially, nurses and pharmacists who were in charge of patients undergoing chemotherapy gave questionnaires to all patients with cancer who were receiving chemotherapy, not just women with breast cancer. Thus, although some items did not apply to breast cancer treatment, those that corresponded to the QOL‐ACD‐B were used as they were. Items with detailed descriptions in the medical record were inferred from sentences and scoring. Subjects that were difficult to evaluate were treated as ‘no answer’. Changes were calculated by evaluating QOL scores before and after POC for each patient, and evaluated in relation to clinical factors and survival.

### Survival

Disease‐free survival (DFS) was defined as the time interval from the date of primary surgery to the date of disease progression and/or recurrence. Overall survival (OS) was defined, in days, as the date of the primary surgery to the date of death. All women were followed up with a physical examination every 3 months, ultrasonography every 6 months, and CT and bone scintigraphy annually.

### Statistical analysis

Statistical analysis was performed using the JMP® 13 software package (SAS Institute, Cary, North Carolina, USA). The relationship between each factor was examined with the χ^2^ test. Distributions of the QOL score before and after POC were expressed in box–whisker plots, with comparisons using Student's *t* test. The Kaplan–Meier method and log rank test were used to compare DFS and OS, and QOL‐ACD‐B scores. Hazard ratios (HRs) and 95 per cent confidence intervals were calculated with the Cox proportional hazards model. Univariable and multivariable analyses were performed using the Cox regression model, with a backward stepwise method used for variable selection in the multivariable analyses. *P* < 0·050 was considered statistically significant, even in univariable and multivariable analysis of prognosis. The cut‐off value for QOL before and after POC was determined by receiver operating characteristic (ROC) curve analysis of events (recurrence or death before recurrence).

## Results

Clinicopathological features of patients included in the study are shown in *Table* 2. Median age at operation was 55 (range 27–90) years and median tumour diameter was 2·9 (1·0–9·8) cm. Thirty‐eight patients (12·7 per cent) had skin infiltration and 210 (70·0 per cent) were diagnosed with lymph node metastasis at the time of breast cancer diagnosis. One hundred and forty‐nine women (49·7 per cent) were diagnosed with HRBC, 57 (19·0 per cent) with HER2BC and 94 (31·3 per cent) with TNBC. The response rate in the study cohort was 89·3 per cent, with 99 women (33·0 per cent) achieving a pCR. The median duration of follow‐up after surgery was 1477 (range 63–3524) days.

**Table 2:** Clinicopathological features of patients treated with preoperative chemotherapy

	No. of patients* (*n* = 300)
Age (years)†	55 (27–90)
Tumour size (cm)†	2·9 (1·0–9·8)
Skin infiltration	
No	262 (87·3)
Yes	38 (12·7)
Lymph node metastasis	
N0	90 (30·0)
N1	116 (38·7)
N2	65 (21·7)
N3	29 (9·7)
Oestrogen receptor status	
Negative	155 (51·7)
Positive	145 (48·3)
Progesterone receptor status	
Negative	200 (66·7)
Positive	100 (33·3)
HER2 status	
Negative	212 (70·7)
Positive	88 (29·3)
Ki67 status	
Negative	96 (32·0)
Positive	204 (68·0)
Intrinsic subtype	
HRBC	149 (49·7)
HER2BC	57 (19·0)
TNBC	94 (31·3)
ORR	
Non‐responder	32 (10·7)
Responder	268 (89·3)
pCR	
No	201 (67·0)
Yes	99 (33·0)
Recurrence	
No	238 (79·3)
Yes	62 (20·7)
Died from breast cancer	
No	270 (90·0)
Yes	30 (10·0)
QOL‐ACD‐B score before POC	
Low	220 (73·3)
High	80 (26·7)
QOL‐ACD‐B score after POC	
Low	248 (82·7)
High	52 (17·3)

* With percentages in parentheses unless indicated otherwise;

† values are median (range). HER, human epidermal growth factor receptor; HRBC, hormone receptor‐positive breast cancer (oestrogen receptor (ER) + and/or progesterone receptor (PgR)+); HER2BC, human epidermal growth factor receptor 2‐enriched breast cancer (ER−, PgR− and HER2+); TNBC, triple‐negative breast cancer (ER−, PgR− and HER2−); ORR, objective response rate; pCR, pathological complete response.

### Comparison of clinicopathological features and QOL‐ACD‐B score

Median QOL before POC was 92·188 (range 64·063–98·438), and the cut‐off value was the same as the median (*Fig. S1A*, supporting information). In addition, median QOL after POC was 82·813 (42·188–96·875), but the cut‐off value was 89·025 (*Fig. S1B*, supporting information).

### Changes in quality‐of‐life scores before and after preoperative chemotherapy

Clinicopathological features and QOL‐ACD‐B scores before and after POC are compared in *Table* 3. Before POC, tumour size was significantly greater, and skin infiltration and lymph node metastasis were observed more frequently in patients with low QOL scores than in those with high scores (*P* = 0·005, *P* < 0·001 and *P* < 0·001 respectively). After POC, the ORR was greater in patients with high QOL scores than in those with low scores (*P* = 0·025). There was no significant difference in QOL scores before and after POC (*P* = 0·766). Before POC, when comparing high and low QOL groups on subscales, the low QOL group had a significantly lower score for Physical symptoms and pain (*P* < 0·001) and Dress, sexual aspect, other categories (*P* < 0·001), whereas Satisfaction with treatment and coping with disease (*P* = 0·443), and Side‐effects of treatment categories showed no change (*P* = 0·253) (*Fig. S2*, supporting information). After POC, there was no significant difference between Physical symptoms and pain, and Dress, sexual aspect, other categories (*P* = 0·114 and *P* = 0·369 respectively) (*Fig. S3*, supporting information).

**Table 3:** Comparison of clinicopathological features and QOL‐ACD‐B score before and after preoperative chemotherapy

	QOL‐ACD‐B score before POC		*P* *	QOL‐ACD‐B score after POC		*P* *
	High (*n* = 80)	Low (*n* = 220)		High (*n* = 52)	Low (*n* = 248)	
Age at operation (years)			0·095			0·355
≤ 55	48 (60)	108 (49·1)		24 (46)	132 (53·2)	
> 55	32 (40)	112 (50·9)		28 (54)	116 (46·8)	
Tumour size (cm)			0·005			0·509
≤ 2·9	51 (64)	100 (45·5)		24 (46)	127 (51·2)	
> 2·9	29 (36)	120 (54·5)		28 (54)	121 (48·8)	
Skin infiltration			< 0·001			0·850
No	79 (99)	183 (83·2)		45 (87)	217 (87·5)	
Yes	1 (1)	37 (16·8)		7 (13)	31 (12·5)	
Lymph node status			< 0·001			0·144
Negative	36 (45)	54 (24·5)		20 (38)	70 (28·2)	
Positive	44 (55)	166 (75·5)		32 (62)	178 (71·8)	
Oestrogen receptor status			0·862			0·239
Negative	42 (53)	113 (51·4)		23 (44)	132 (53·2)	
Positive	38 (47)	107 (48·6)		29 (56)	116 (46·8)	
Progesterone receptor status			0·646			0·390
Negative	55 (69)	145 (65·9)		32 (62)	168 (67·7)	
Positive	25 (31)	75 (34·1)		20 (38)	80 (32·3)	
HER2 status			0·481			0·733
Negative	59 (74)	153 (69·5)		41 (79)	171 (69·0)	
Positive	21 (26)	67 (30·5)		11 (21)	77 (31·0)	
Ki67 status			0·469			0·274
Negative	23 (29)	73 (33·2)		20 (38)	76 (30·6)	
Positive	57 (71)	147 (66·8)		32 (62)	172 (69·4)	
Intrinsic subtype HRBC			0·945			0·204
No	40 (50)	111 (50·5)		22 (42)	129 (52·0)	
Yes	40 (50)	109 (49·5)		30 (58)	119 (48·0)	
Intrinsic subtype HER2BC			0·466			0·733
No	67 (84)	176 (80·0)		43 (83)	200 (80·6)	
Yes	13 (16)	44 (20·0)		9 (17)	48 (19·4)	
Intrinsic subtype TNBC			0·588			0·280
No	53 (66)	153 (69·5)		39 (75)	167 (67·3)	
Yes	27 (34)	67 (30·5)		13 (25)	81 (32·7)	
ORR			0·822			0·025
Non‐responder	8 (10)	24 (10·9)		1 (2)	31 (12·5)	
Responder	72 (90)	196 (89·1)		51 (98)	217 (87·5)	
Pathological response			0·658			0·708
Not pCR	52 (65)	149 (67·7)		36 (69)	165 (66·5)	
pCR	28 (35)	71 (32·3)		16 (31)	83 (33·5)	
QOL‐ACD‐B score after POC			0·766			
Low	67 (84)	181 (82·3)		–	–	
High	13 (16)	39 (17·7)		–	–	

Values in parentheses are percentages. POC, preoperative chemotherapy; HER, human epidermal growth factor receptor; HRBC, hormone receptor‐positive breast cancer (oestrogen receptor (ER) + and/or progesterone receptor (PgR)+); HER2BC; human epidermal growth factor receptor 2‐enriched breast cancer (ER−, PgR– and HER2+); TNBC, triple‐negative breast cancer (ER–, PgR– and HER2–); ORR, objective response rate; pCR, pathological complete response.

* χ^2^ test.

**Figure 1:**
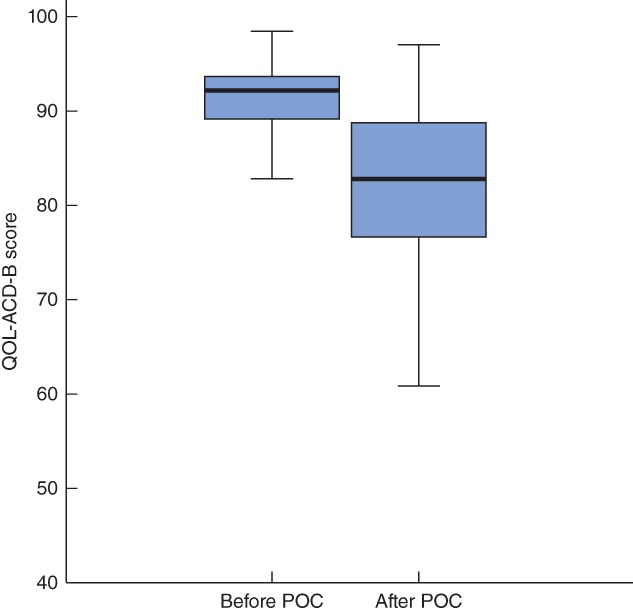
QOL‐ACD‐B score before and after preoperative chemotherapy. Median values, interquartile ranges and ranges are denoted by horizontal bars, boxes and error bars respectively. POC, preoperative chemotherapy. *P* < 0·001 (Student's *t* test)

Although there was no significant difference between each QOL group before and after POC, comparison of all QOL groups showed a significant decrease in QOL after POC (*P* < 0·001) (*Fig*. 1). When subscale score changes before and after POC were examined, there was a significant decrease in Physical symptoms and pain, and Side‐effects of treatment (both *P* < 0·001) (*Fig. S4A,C*, supporting information). Satisfaction with treatment and coping with disease showed no change (*P* = 0·725) (*Fig. S4B*, supporting information), whereas Dress, sexual aspect, other showed a significant increase (*P* < 0·001) (*Fig. S4D*, supporting information).

### Comparison of QOL‐ACD‐B scores with disease‐free and overall survival

The cut‐off value for the QOL‐ACD‐B score contributing to DFS was calculated from ROC analysis, yielding a distribution of 80 patients (26·7 per cent) in the high QOL group and 220 (73·3 per cent) in the low QOL group before POC (area under the receiver operating characteristic (ROC) curve (AUC) 0·674, 95 per cent c.i. 0·599 to 0·748, *P* < 0·001; sensitivity 72·7 per cent, specificity 46·9 per cent) (*Fig. S1A*, supporting information). After POC, there were 52 patients (17·3 per cent) in the high QOL group and 248 (82·7 per cent) in the low QOL group (AUC 0·607, 0·529 to 0·684, *P* = 0·010; sensitivity 37·5 per cent, specificity 15·6 per cent) (*Fig. S1B*, supporting information).

**Figure 2:**
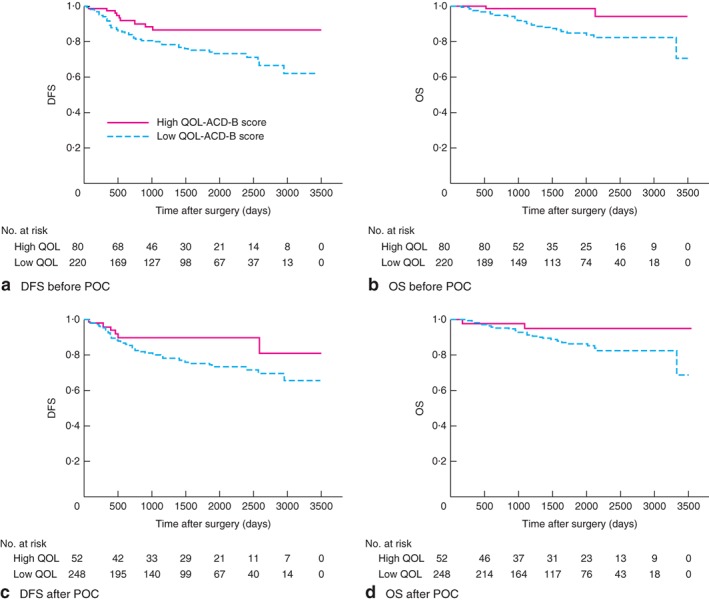
Disease‐free and overall survival in women with high and low QOL‐ACD‐B scores before and after postoperative chemotherapy. **a,c** Disease‐free survival (DFS) and **b,d** overall survival (OS) in women with high and low QOL‐ACD‐B scores **a,b** before and **c,d** after postoperative chemotherapy (POC). **a** *P* = 0·025, **b** *P* = 0·018, **c** *P* = 0·066, **d** *P* = 0·079 (log rank test)

Before POC, high QOL score was significantly associated with better survival, in terms of both DFS (*P* = 0·025) and OS (*P* = 0·018) (*Fig*. 2 *a,b*). After POC, there was no significant difference in DFS or OS in patients with high and low QOL scores (*Fig*. 2 *c,d*). In univariable analysis, a high QOL score before POC or after POC was associated with longer DFS (before POC: HR 0·45, 95 per cent c.i. 0·21 to 0·87, *P* = 0·017; after POC: HR 0·46, 0·18 to 0·99, *P* = 0·047). In multivariable analysis, however, a high QOL score before POC (HR 0·52, 0·23 to 1·05; *P* = 0·070) or after POC (HR 0·54, 0·20 to 1·20; *P* = 0·135) was not an independent factor for DFS (*Table* 4). In univariable analysis of OS, a high QOL score before POC (HR 0·21, 0·03 to 0·69; *P* = 0·007) and after POC (HR 0·30, 0·05 to 0·99; *P* = 0·048) was associated with longer survival, and was an independent factor in multivariable analysis (HR 0·26, 0·04 to 0·96; *P* = 0·042) (*Table* 5). No QOL subscale category was a significant predictor of prognosis (*Tables S1* and *S2*, supporting information).

**Table 4:** Univariable and multivariable analysis of disease‐free survival in 300 patients treated with preoperative chemotherapy

	Univariable analysis		Multivariable analysis	
	Hazard ratio	*P*	Hazard ratio	*P*
Age at operation (years)				
≤ 55	0·69 (0·41, 1·14)	0·151	0·64 (0·37, 1·08)	0·094
> 55	1·00 (reference)		1·00 (reference)	
Tumour size (cm)				
≤ 2·9	1·30 (0·79, 2·15)	0·306	0·78 (0·45, 1·36)	0·375
> 2·9	1·00 (reference)		1·00 (reference)	
Skin infiltration				
No	2·03 (1·06, 3·65)	0·035	2·43 (1·17, 4·78)	0·018
Yes	1·00 (reference)		1·00 (reference)	
Lymph node status				
Negative	2·43 (1·26, 5·27)	0·007	2·19 (1·08, 4·97)	0·030
Positive	1·00 (reference)		1·00 (reference)	
Oestrogen receptor status				
Negative	0·75 (0·45, 1·24)	0·262	0·17 (0·05, 0·59)	0·005
Positive	1·00 (reference)		1·00 (reference)	
Progesterone receptor status				
Negative	0·93 (0·54, 1·55)	0·781	1·20 (0·56, 2·69)	0·645
Positive	1·00 (reference)		1·00 (reference)	
HER2 status				
Negative	0·59 (0·30, 1·06)	0·080	0·26 (0·07, 0·78)	0·014
Positive	1·00 (reference)		1·00 (reference)	
Ki67 status				
Negative	0·94 (0·56, 1·63)	0·830	1·10 (0·62, 1·99)	0·755
Positive	1·00 (reference)		1·00 (reference)	
Intrinsic subtype TNBC				
No	1·53 (0·91, 2·54)	0·109	0·32 (0·08, 1·18)	0·087
Yes	1·00 (reference)		1·00 (reference)	
ORR				
Non‐responder	0·27 (0·15, 0·49)	< 0·001	0·20 (0·10, 0·39)	< 0·001
Responder	1·00 (reference)		1·00 (reference)	
Pathological response				
Not pCR	0·44 (0·22, 0·80)	0·006	0·44 (0·21, 0·88)	0·020
pCR	1·00 (reference)		1·00 (reference)	
QOL‐ACD‐B score before POC				
Low	0·45 (0·21, 0·87)	0·017	0·52 (0·23, 1·05)	0·070
High	1·00 (reference)		1·00 (reference)	
QOL‐ACD‐B score after POC				
Low	0·46 (0·18, 0·99)	0·047	0·54 (0·20, 1·20)	0·135
High	1·00 (reference)		1·00 (reference)	

Values in parentheses are 95 per cent confidence intervals. HER, human epidermal growth factor receptor; TNBC, triple‐negative breast cancer; ORR, objective response rate; pCR, pathological complete response; POC, preoperative chemotherapy.

**Table 5:** Univariable and multivariable analysis of overall survival in 300 patients treated with preoperative chemotherapy

	Univariable analysis		Multivariable analysis	
	Hazard ratio	*P*	Hazard ratio	*P*
Age at operation (years)				
≤ 55	0·66 (0·31, 1·37)	0·268	0·79 (0·36, 1·69)	0·546
> 55	1·00 (reference)		1·00 (reference)	
Tumour size (cm)				
≤ 2·9	1·13 (0·59, 2·53)	0·591	0·88 (0·37, 2·03)	0·762
> 2·9	1·00 (reference)		1·00 (reference)	
Skin infiltration				
No	2·23 (0·88, 4·97)	0·086	2·66 (0·92, 7·29)	0·071
Yes	1·00 (reference)		1·00 (reference)	
Lymph node status				
Negative	3·30 (1·16, 13·82)	0·022	2·17 (0·69, 9·87)	0·203
Positive	1·00 (reference)		1·00 (reference)	
Oestrogen receptor status				
Negative	0·48 (0·21, 1·00)	0·050	0·06 (0·01, 0·38)	0·004
Positive	1·00 (reference)		1·00 (reference)	
Progesterone receptor status				
Negative	0·88 (0·39, 1·84)	0·742	2·52 (0·63, 12·49)	0·201
Positive	1·00 (reference)		1·00 (reference)	
HER2 status				
Negative	0·29 (0·07, 0·82)	0·017	0·09 (0·01, 0·64)	0·013
Positive	1·00 (reference)		1·00 (reference)	
Ki67 status				
Negative	1·43 (0·66, 3·43)	0·374	1·26 (0·52, 3·27)	0·620
Positive	1·00 (reference)		1·00 (reference)	
Intrinsic subtype TNBC				
No	2·85 (1·37, 6·03)	0·005	0·26 (0·03, 2·40)	0·239
Yes	1·00 (reference)		1·00 (reference)	
ORR				
Non‐responder	0·23 (0·10, 0·55)	0·002	0·20 (0·08, 0·53)	0·002
Responder	1·00 (reference)		1·00 (reference)	
Pathological response				
Not pCR	0·38 (0·13, 0·91)	0·028	0·38 (0·11, 1·06)	0·066
pCR	1·00 (reference)		1·00 (reference)	
QOL‐ACD‐B score before POC				
Low	0·21 (0·03, 0·69)	0·007	0·26 (0·04, 0·96)	0·042
High	1·00 (reference)		1·00 (reference)	
QOL‐ACD‐B score after POC				
Low	0·30 (0·05, 0·99)	0·048	0·34 (0·05, 1·26)	0·116
High	1·00 (reference)		1·00 (reference)	

Values in parentheses are 95 per cent confidence intervals. HER, human epidermal growth factor receptor; TNBC, triple‐negative breast cancer; ORR, objective response rate; pCR, pathological complete response; POC, preoperative chemotherapy.

## Discussion

Reports that patients' QOL has an influence on cancer treatment are increasing. The European Organization for Research and Treatment of Cancer Quality of Life Questionnaire – Core 30 (EORTC QLQ‐C30), Functional Assessment of Cancer Therapy (FACT) and Cancer Rehabilitation Evaluation System (CARES) are used to assess QOL for various cancers^16^, ^17^, ^18^, ^19^. This study used the Japanese QOL‐ACD‐B questionnaire, which is a specific QOL scale for breast cancer^7^ ^8^.

In the present study, patients with a low QOL‐ACD‐B score before POC had worse DFS and OS than those with high scores. In subscale analysis, scores were influenced mainly the categories by Physical symptoms and pain, and Dress, sexual aspect, other.

When examining the relationship with clinical features, in patients with a low QOL score before POC, tumour size was significantly greater, and both skin infiltration and lymph node metastasis were observed with a higher frequency.

QOL‐ACD‐B scores fell significantly after administration of POC. Side‐effects such as hair loss, fatigue, numbness and taste disorder resulting from POC treatment may all have affected QOL‐ACD‐B scores. Chee Chean and colleagues^20^ examined QOL after the treatment of breast cancer with anticancer drugs and reported that age, stage and co‐morbidity showed no clear association with global health status. In the present study, there was no significant difference between QOL scores before and after POC, and the difference in Physical symptoms and pain, and Dress, sexual aspect, other subscale category scores seen before POC was not apparent after POC, probably reflecting tumour shrinkage in patients who initially experienced pain and skin ulceration, with improved QOL due to the disappearance of breast cancer symptoms. One study^21^ found no significant difference in physical symptoms or functional aspects between older and young women, although younger patients experienced a significant decrease in QOL; however, the present study found no influence related to age.

In terms of the relationship between QOL and prognosis, some studies^22^ ^23^ found both QOL score and QOL score change after treatment to be significant predictors of subsequent patient survival. Furthermore, several studies^24^, ^25^, ^26^, ^27^, ^28^ have reported that appetite loss and pain are independent prognostic factors of QOL measures. The present study also analysed the change in QOL before and after POC, but this was not a significant predictor of prognosis (data not shown). Although the QOL‐ACD‐B does include many items of physical QOL, there are few mental or social QOL items. Previous reports^29^, ^30^, ^31^ have shown improvement in both QOL and prognosis with the early use of psychological care and palliative treatment. By changing the items of QOL evaluation, such as increasing the number of mental or social QOL items, it may be possible to show that the change in QOL affects prognosis.

The main limitation of this study is its retrospective design, where QOL was evaluated from information obtained from retrieved medical records. There are, however, relatively few interventional studies that have evaluated QOL as in previous studies^29^, ^30^, ^31^. To evaluate QOL with greater precision, a prospective study is warranted. The present study might also be considered a reference for setting new evaluation items, in addition to those in the existing questionnaire.

## Supporting information


**Table S1** Univariable and multivariable analysis of disease‐free survival in patients treated with preoperative chemotherapy
**Table S2** Univariable and multivariable analysis of overall survival in patients treated with preoperative chemotherapy
**Fig. S1** Receiver operating characteristic (ROC) curve analysis. The cut‐off value of QOL‐ACD‐B contributing to DFS was calculated from ROC analysis, yielding a distribution of 80 patients in the high QOL group and 220 patients in the low QOL group before POC treatment (AUC: 0·674, p < 0·001, 95% CI: 0·599–0·748, sensitivity = 72·7%, specificity = 46·9%) **(A)**. After POC treatment, 52 patients were included in the high QOL group and 248 patients in the low QOL group (AUC: 0·607, p = 0·010, 95% CI: 0·529–0·684, sensitivity = 37·5%, specificity = 15·6%) **(B)**.
**Fig. S2** Comparison of high and low QOL groups on a subscale before POC. Before POC, when comparing high and low QOL groups on a subscale, the low QOL group was significantly lower in the “Physical symptoms and pain” (p < 0·01) **(A)**, and “Dress, sexual aspect, other” categories (p < 0·01) **(D)**, while “Satisfaction with treatment and coping with disease” (p = 0·44) **(B)** and “Side‐effects of treatment” showed no change (p = 0·25) **(C)**. Where the box covers the bar for the median value, the position of the bar is indicated by an arrow
**Fig. S3** Comparison of subscales after POC in groups with high and low QOL before POC. After POC, when comparing high and low QOL groups before POC on a subscale, the low QOL group was significantly lower in the “Satisfaction with treatment and coping with disease” (p = 0·01) **(B)**, and “Side‐effects of treatment” categories (p = 0·04) **(C)**, while “Physical symptoms and pain” (p = 0·11) **(A)** and “Dress, sexual aspect, other” showed no change (p = 0·37) **(D)**.**)**. Where the box covers the bar for the median value, the position of the bar is indicated by an arrow
**Fig. S4** Changes in subscale QOL scores before and after POC. Each subscale QOL score before and after POC is showed by box‐whisker plot diagram. There was a significant decrease in “Physical symptoms and pain” and “Side‐effects of treatment” (p < 0·001) **(A)**, (p < 0·001) **(C)**, and “Satisfaction with treatment and coping with disease” showed no change (p = 0·725) **(B)**, while “Dress, sexual aspect, other” showed a significant increase (p < 0·001) **(D)**.**)**. Where the box covers the bar for the median value, the position of the bar is indicated by an arrow

## References

[1] Howell D , Molloy S , Wilkinson K , Green E , Orchard K , Wang K *et al* Patient‐reported outcomes in routine cancer clinical practice: a scoping review of use, impact on health outcomes, and implementation factors. Ann Oncol 2015; 26: 1846–1858. 25888610 10.1093/annonc/mdv181

[2] World Health Organization . Basic Documents (39th edn). WHO: Geneva, 1992.

[3] Maisey NR , Norman A , Watson M , Allen MJ , Hill ME , Cunningham D. Baseline quality of life predicts survival in patients with advanced colorectal cancer. Eur J Cancer 2002; 38: 1351–1357. 12091066 10.1016/s0959-8049(02)00098-9

[4] Kaasa S , Mastekaasa A , Lund E . Prognostic factors for patients with inoperable non‐small cell lung cancer, limited disease. The importance of patients' subjective experience of disease and psychosocial well‐being. Radiother Oncol 1989; 15: 235–242. 2549582 10.1016/0167-8140(89)90091-1

[5] Gotay CC , Kawamoto CT , Bottomley A , Efficace F . The prognostic significance of patient‐reported outcomes in cancer clinical trials. J Clin Oncol 2008; 26: 1355–1363. 18227528 10.1200/JCO.2007.13.3439

[6] Dancey J , Zee B , Osoba D , Whitehead M , Lu F , Kaizer L *et al* Quality of life scores: an independent prognostic variable in a general population of cancer patients receiving chemotherapy. The National Cancer Institute of Canada Clinical Trials Group. Qual Life Res 1997; 6: 151–158. 9161115 10.1023/a:1026442201191

[7] Kurihara M , Shimizu H , Tsuboi K , Kobayashi K , Murakami M , Eguchi K *et al* Development of quality of life questionnaire in Japan: quality of life assessment of cancer patients receiving chemotherapy. Psychooncology 1999; 8: 355–363. 10474853 10.1002/(SICI)1099-1611(199907/08)8:4<355::AID-PON401>3.0.CO;2-I

[8] Otsuka S , Watanabe N , Sasaki Y , Shimojima R . Postoperative courses of breast reconstruction using inferior adipofascial tissue repair. Breast Cancer 2015; 22: 570–577. 24925523 10.1007/s12282-014-0522-6

[9] McShane LM , Altman DG , Sauerbrei W , Taube SE , Gion M , Clark GM ; Statistics Subcommittee of the NCI‐EORTC Working Group on Cancer Diagnostics . Reporting recommendations for tumor marker prognostic studies. J Clin Oncol 2005; 23: 9067–9072. 16172462 10.1200/JCO.2004.01.0454

[10] Mauri D , Pavlidis N , Ioannidis JP . Neoadjuvant *versus* adjuvant systemic treatment in breast cancer: a meta‐analysis. J Natl Cancer Inst 2005; 97: 188–194. 15687361 10.1093/jnci/dji021

[11] Mieog JS , van der Hage JA , van de Velde CJ . Preoperative chemotherapy for women with operable breast cancer. Cochrane Database Syst Rev 2007; (2)CD005002. 10.1002/14651858.CD005002.pub2 PMC7388837 17443564

[12] Kawajiri H , Takashima T , Onoda N , Kashiwagi S , Noda S , Ishikawa T *et al* Efficacy and feasibility of neoadjuvant chemotherapy with FEC 100 followed by weekly paclitaxel for operable breast cancer. Oncol Lett 2012; 4: 612–616. 23205071 10.3892/ol.2012.801 PMC3506652

[13] Eisenhauer EA , Therasse P , Bogaerts J , Schwartz LH , Sargent D , Ford R *et al* New response evaluation criteria in solid tumours: revised RECIST guideline (version 1·1). Eur J Cancer 2009; 45: 228–247. 19097774 10.1016/j.ejca.2008.10.026

[14] Kashiwagi S , Onoda N , Asano Y , Kurata K , Morisaki T , Noda S *et al* Partial mastectomy using manual blunt dissection (MBD) in early breast cancer. BMC Surg 2015; 15: 117. 26494510 10.1186/s12893-015-0102-5 PMC4618878

[15] Wolmark N , Wang J , Mamounas E , Bryant J , Fisher B. Preoperative chemotherapy in patients with operable breast cancer: nine‐year results from National Surgical Adjuvant Breast and Bowel Project B‐18. J Natl Cancer Inst Monogr 2001; 30: 96–102. 10.1093/oxfordjournals.jncimonographs.a003469 11773300

[16] Cella DF , Tulsky DS , Gray G , Sarafian B , Linn E , Bonomi A *et al* The Functional Assessment of Cancer Therapy scale: development and validation of the general measure. J Clin Oncol 1993; 11: 570–579. 8445433 10.1200/JCO.1993.11.3.570

[17] Aaronson NK , Ahmedzai S , Bergman B , Bullinger M , Cull A , Duez NJ *et al* The European Organization for Research and Treatment of Cancer QLQ‐C30: a quality‐of‐life instrument for use in international clinical trials in oncology. J Natl Cancer Inst 1993; 85: 365–376. 8433390 10.1093/jnci/85.5.365

[18] Schouten B , Hellings J , Vankrunkelsven P , Mebis J , Bulens P , Buntinx F *et al* Qualitative research on the Belgian Cancer Rehabilitation Evaluation System (CARES): an evaluation of the content validity and feasibility. J Eval Clin Pract 2017; 23: 599–607. 28111884 10.1111/jep.12681

[19] Schouten B , Hellings J , Van Hoof E , Vankrunkelsven P , Bulens P , Buntinx F *et al* Validation of the Flemish CARES, a quality of life and needs assessment tool for cancer care. BMC Cancer 2016; 16: 696. 27576341 10.1186/s12885-016-2728-9 PMC5006609

[20] Chee Chean D , Kuo Zang W , Lim M , Zulkefle N . Health related quality of life (HRQoL) among breast cancer patients receiving chemotherapy in Hospital Melaka: single centre experience. Asian Pac J Cancer Prev 2016; 17: 5121–5126. 28122444 10.22034/APJCP.2016.17.12.5121 PMC5454646

[21] Wenzel LB , Fairclough DL , Brady MJ , Cella D , Garrett KM , Kluhsman BC *et al* Age‐related differences in the quality of life of breast carcinoma patients after treatment. Cancer 1999; 86: 1768–1774. 10547550

[22] Coates A , Gebski V , Signorini D , Murray P , McNeil D , Byrne M *et al* Prognostic value of quality‐of‐life scores during chemotherapy for advanced breast cancer. Australian New Zealand Breast Cancer Trials Group. J Clin Oncol 1992; 10: 1833–1838. 1453197 10.1200/JCO.1992.10.12.1833

[23] Shimozuma K , Sonoo H , Ichihara K , Tanaka K . The prognostic value of quality‐of‐life scores: preliminary results of an analysis of patients with breast cancer. Surg Today 2000; 30: 255–261. 10752779 10.1007/s005950050055

[24] Efficace F , Biganzoli L , Piccart M , Coens C , Van Steen K , Cufer T *et al*; EORTC‐BCG‐IDBBC‐NDDG . Baseline health‐related quality‐of‐life data as prognostic factors in a phase III multicentre study of women with metastatic breast cancer. Eur J Cancer 2004; 40: 1021–1030. 15093577 10.1016/j.ejca.2004.01.014

[25] Efficace F , Therasse P , Piccart MJ , Coens C , van Steen K , Welnicka‐Jaskiewicz M *et al* Health‐related quality of life parameters as prognostic factors in a nonmetastatic breast cancer population: an international multicenter study. J Clin Oncol 2004; 22: 3381–3388. 15310784 10.1200/JCO.2004.02.060

[26] Kramer JA , Curran D , Piccart M , de Haes JC , Bruning P , Klijn J *et al* Identification and interpretation of clinical and quality of life prognostic factors for survival and response to treatment in first‐line chemotherapy in advanced breast cancer. Eur J Cancer 2000; 36: 1498–1506. 10930797 10.1016/s0959-8049(00)00144-1

[27] Luoma ML , Hakamies‐Blomqvist L , Sjöström J , Pluzanska A , Ottoson S , Mouridsen H *et al* Prognostic value of quality of life scores for time to progression (TTP) and overall survival time (OS) in advanced breast cancer. Eur J Cancer 2003; 39: 1370–1376. 12826039 10.1016/s0959-8049(02)00775-x

[28] Lee CK , Stockler MR , Coates AS , Gebski V , Lord SJ , Simes RJ ; Australian New Zealand Breast Cancer Trials Group . Self‐reported health‐related quality of life is an independent predictor of chemotherapy treatment benefit and toxicity in women with advanced breast cancer. Br J Cancer 2010; 102: 1341–1347. 20389302 10.1038/sj.bjc.6605649 PMC2865758

[29] Spiegel D , Bloom JR , Kraemer HC , Gottheil E . Effect of psychosocial treatment on survival of patients with metastatic breast cancer. Lancet 1989; 2: 888–891. 2571815 10.1016/s0140-6736(89)91551-1

[30] Spiegel D , Sephton SE , Terr AI , Stites DP . Effects of psychosocial treatment in prolonging cancer survival may be mediated by neuroimmune pathways. Ann N Y Acad Sci 1998; 840: 674–683. 9629294 10.1111/j.1749-6632.1998.tb09606.x

[31] Küchler T , Bestmann B , Rappat S , Henne‐Bruns D , Wood‐Dauphinee S . Impact of psychotherapeutic support for patients with gastrointestinal cancer undergoing surgery: 10‐year survival results of a randomized trial. J Clin Oncol 2007; 25: 2702–2708. 17602075 10.1200/JCO.2006.08.2883

