# Supplementary material for: Prognostic value of quality‐of‐life scores in patients with breast cancer undergoing preoperative chemotherapy
Source: BJS Open. 2018 Nov 26;3(1):38–47. doi: 10.1002/bjs5.50108 (PMC6354182; doi:10.1002/bjs5.50108)
Supplement: Supplementary file 1 — Table S1 Univariable and multivariable analysis of disease‐free survival in patients treated with preoperative chemotherapy Table S2 Univariable and multivariable analysis of overall survival in patients treated with preoperative chemotherapy Fig. S1 Receiver operating characteristic (ROC) curve analysis. The cut‐off value of QOL‐ACD‐B contributing to DFS was calculated from ROC analysis, yielding a distribution of 80 patients in the high QOL group and 220 patients in the low QOL group before POC treatment (AUC: 0·674, p < 0·001, 95% CI: 0·599–0·748, sensitivity = 72·7%, specificity = 46·9%) (A). After POC treatment, 52 patients were included in the high QOL group and 248 patients in the low QOL group (AUC: 0·607, p = 0·010, 95% CI: 0·529–0·684, sensitivity = 37·5%, specificity = 15·6%) (B). Fig. S2 Comparison of high and low QOL groups on a subscale before POC. Before POC, when comparing high and low QOL groups on a subscale, the low QOL group was significantly lower in the “Physical symptoms and pain” (p < 0·01) (A), and “Dress, sexual aspect, other” categories (p < 0·01) (D), while “Satisfaction with treatment and coping with disease” (p = 0·44) (B) and “Side‐effects of treatment” showed no change (p = 0·25) (C). Where the box covers the bar for the median value, the position of the bar is indicated by an arrow Fig. S3 Comparison of subscales after POC in groups with high and low QOL before POC. After POC, when comparing high and low QOL groups before POC on a subscale, the low QOL group was significantly lower in the “Satisfaction with treatment and coping with disease” (p = 0·01) (B), and “Side‐effects of treatment” categories (p = 0·04) (C), while “Physical symptoms and pain” (p = 0·11) (A) and “Dress, sexual aspect, other” showed no change (p = 0·37) (D).). Where the box covers the bar for the median value, the position of the bar is indicated by an arrow Fig. S4 Changes in subscale QOL scores before and after POC. Each subscale QOL score before and after POC [file BJS5-3-38-s001.docx]

**BJS5_50108**

**Prognostic value of quality-of-life scores in patients with breast cancer undergoing preoperative chemotherapy**

**K. Takada, S. Kashiwagi, Y. Fukui, W. Goto, Y. Asano, T. Morisaki, T. Takashima, K. Hirakawa and M. Ohira**

**Table S1** Univariable and multivariable analysis of disease-free survival in patients treated with preoperative chemotherapy

|  | Univariable analysis | | |  | Multivariable analysis | | |
| --- | --- | --- | --- | --- | --- | --- | --- |
| Parameters | Hazard ratio | 95% CI | *p* value |  | Hazard ratio | 95% CI | *p* value |
| Age at operation (yr)  ≤ 55  > 55 | 0.693 | 0.414-1.143 | 0.151 |  | 0.610 | 0.352-1.041 | 0.070 |
| Tumour size (cm)  ≤ 2.9  > 2.9 | 1.297 | 0.788-2.154 | 0.306 |  | 0.780 | 0.437-1.394 | 0.399 |
| Skin infiltration  Negative  Positive | 2.034 | 1.056-3.646 | 0.035 |  | 2.852 | 1.300-6.069 | 0.010 |
| Lymph node status  Negative  Positive | 2.426 | 1.259-5.268 | 0.007 |  | 2.192 | 1.045-5.125 | 0.037 |
| Oestrogen receptor  Negative  Positive | 0.751 | 0.451-1.238 | 0.262 |  | 0.156 | 0.042-0.558 | 0.004 |
| Progesterone receptor  Negative  Positive | 0.928 | 0.539-1.553 | 0.781 |  | 1.228 | 0.555-2.831 | 0.617 |
| HER2  Negative  Positive | 0.586 | 0.298-1.063 | 0.080 |  | 0.277 | 0.075-0.844 | 0.022 |
| Ki67  Negative  Positive | 0.944 | 0.563-1.629 | 0.830 |  | 1.077 | 0.604-1.959 | 0.804 |
| Intrinsic subtype TNBC  No  Yes | 1.532 | 0.907-2.539 | 0.109 |  | 0.333 | 0.083-1.242 | 0.103 |
| ORR  Non-Responders  Responders | 0.265 | 0.151-0.492 | <0.001 |  | 0.180 | 0.090-0.369 | <0.001 |
| Pathological response  Non-pCR  pCR | 0.438 | 0.222-0.795 | 0.006 |  | 0.411 | 0.193-0.828 | 0.012 |
| QOL-ACD-B before POC  Low  High | 0.454 | 0.209-0.874 | 0.017 |  | 0.514 | 0.218-1.104 | 0.090 |
| “Physical symptoms and pain” before POC  Low  High | 0.786 | 0.475-1.295 | 0.343 |  | 0.954 | 0.431-2.190 | 0.909 |
| “Satisfaction with treatment and coping with disease” before POC  Low  High | 0.916 | 0.548-1.511 | 0.733 |  | 0.678 | 0.325-1.452 | 0.312 |
| “Side-effects of treatment” before POC  Low  High | - | - | 0.306 |  | - | - | 0.318 |
| “Dress, sexual aspect, other” before POC  Low  High | 0.756 | 0.313-1.556 | 0.472 |  | 0.972 | 0.384-2.148 | 0.948 |
| QOL-ACD-B after POC  Low  High | 0.462 | 0.178-0.989 | 0.047 |  | 0.824 | 0.253-2.315 | 0.726 |
| “Physical symptoms and pain” after POC  Low  High | 0.932 | 0.564-1.538 | 0.780 |  | 0.798 | 0.453-1.391 | 0.428 |
| “Satisfaction with treatment and coping with disease” after POC  Low  High | 0.627 | 0.241-1.344 | 0.248 |  | 0.942 | 0.345-2.170 | 0.897 |
| “Side-effects of treatment” after POC  Low  High | 0.840 | 0.494-1.395 | 0.505 |  | 1.153 | 0.644-2.012 | 0.625 |
| “Dress, sexual aspect, other” after POC  Low  High | 0.404 | 0.098-1.091 | 0.078 |  | 0.413 | 0.088-1.446 | 0.176 |

POC: preoperative chemotherapy. CI: confidence intervals. HER: human epidermal growth factor receptor. TNBC: triple negative breast cancer (ER-, PgR-, and HER2-). ORR: objective response rate. pCR: pathological complete response.

**Table S2** Univariable and multivariable analysis of overall survival in patients treated with preoperative chemotherapy

|  | Univariable analysis | | |  | Multivariable analysis | | |
| --- | --- | --- | --- | --- | --- | --- | --- |
| Parameters | Hazard ratio | 95% CI | *p* value |  | Hazard ratio | 95% CI | *p* value |
| Age at operation (yr)  ≤ 55  > 55 | 0.664 | 0.311-1.367 | 0.268 |  | 0.749 | 0.330-1.647 | 0.474 |
| Tumour size (cm)  ≤ 2.9  > 2.9 | 1.127 | 0.593-2.528 | 0.591 |  | 0.786 | 0.312-1.936 | 0.600 |
| Skin infiltration  Negative  Positive | 2.233 | 0.883-4.969 | 0.086 |  | 2.672 | 0.827-8.539 | 0.099 |
| Lymph node status  Negative  Positive | 3.299 | 1.164-13.822 | 0.022 |  | 1.926 | 0.581-8.993 | 0.306 |
| Oestrogen receptor  Negative  Positive | 0.477 | 0.213-1.000 | 0.050 |  | 0.038 | 0.005-0.320 | 0.003 |
| Progesterone receptor  Negative  Positive | 0.881 | 0.394-1.843 | 0.742 |  | 3.470 | 0.780-20.544 | 0.106 |
| HER2  Negative  Positive | 0.290 | 0.069-0.822 | 0.017 |  | 0.101 | 0.008-0.814 | 0.029 |
| Ki67  Negative  Positive | 1.430 | 0.662-3.427 | 0.374 |  | 1.085 | 0.436-2.892 | 0.863 |
| Intrinsic subtype TNBC  No  Yes | 2.850 | 1.372-6.028 | 0.005 |  | 0.279 | 0.024-2.856 | 0.289 |
| ORR  Non-Responders  Responders | 0.227 | 0.103-0.548 | 0.002 |  | 0.117 | 0.039-0.363 | <0.001 |
| Pathological response  Non-pCR  pCR | 0.376 | 0.127-0.905 | 0.028 |  | 0.362 | 0.107-1.031 | 0.057 |
| QOL-ACD-B before POC  Low  High | 0.208 | 0.033-0.691 | 0.007 |  | 0.326 | 0.047-1.295 | 0.120 |
| “Physical symptoms and pain” before POC  Low  High | 0.710 | 0.341-1.459 | 0.349 |  | 0.493 | 0.148-1.797 | 0.274 |
| “Satisfaction with treatment and coping with disease” before POC  Low  High | 0.784 | 0.360-1.623 | 0.517 |  | 0.372 | 0.121-1.202 | 0.097 |
| “Side-effects of treatment” before POC  Low  High | - | - | 0.534 |  | - | - | 0.685 |
| “Dress, sexual aspect, other” before POC  Low  High | 0.249 | 0.014-1.174 | 0.087 |  | 0.267 | 0.015-1.374 | 0.130 |
| QOL-ACD-B after POC  Low  High | 0.297 | 0.048-0.992 | 0.048 |  | 0.717 | 0.087-3.778 | 0.120 |
| “Physical symptoms and pain” after POC  Low  High | 0.683 | 0.325-1.402 | 0.299 |  | 0.480 | 0.194-1.130 | 0.093 |
| “Satisfaction with treatment and coping with disease” after POC  Low  High | 0.735 | 0.175-2.089 | 0.599 |  | 1.455 | 0.313-4.938 | 0.595 |
| “Side-effects of treatment” after POC  Low  High | 1.164 | 0.560-2.393 | 0.680 |  | 1.280 | 0.534-3.012 | 0.574 |
| “Dress, sexual aspect, other” after POC  Low  High | 0.596 | 0.096-1.983 | 0.446 |  | 0.258 | 0.033-1.367 | 0.115 |

POC: preoperative chemotherapy. CI: confidence intervals. HER: human epidermal growth factor receptor. TNBC: triple negative breast cancer (ER-, PgR-, and HER2-). ORR: objective response rate. pCR: pathological complete response.


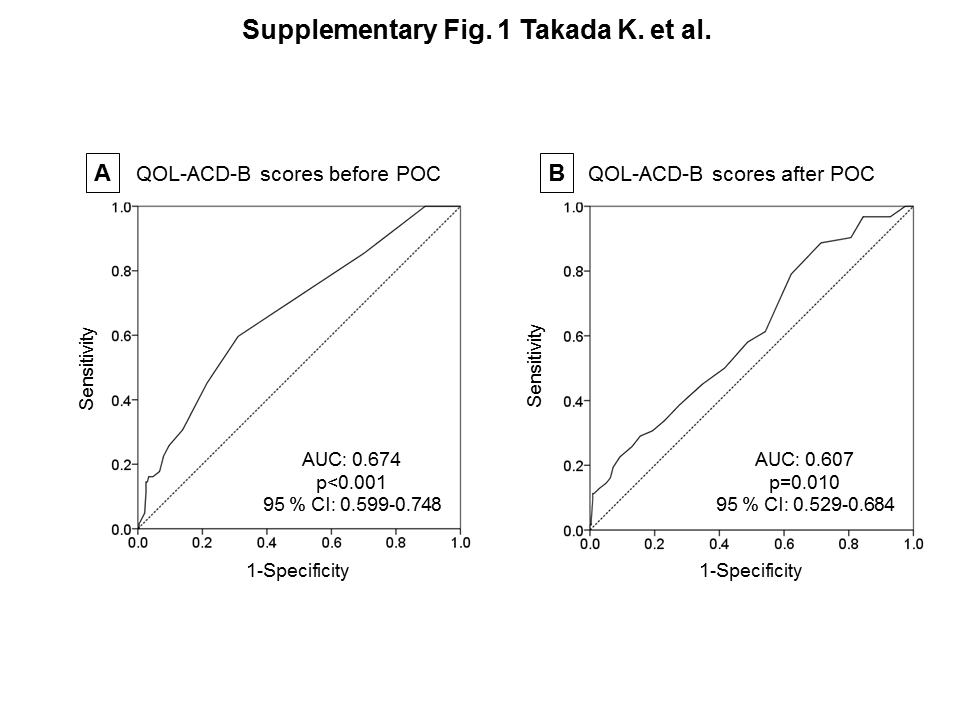


**Fig. S1** Receiver operating characteristic (ROC) curve analysis. The cut-off value of QOL-ACD-B contributing to DFS was calculated from ROC analysis, yielding a distribution of 80 patients in the high QOL group and 220 patients in the low QOL group before POC treatment (AUC: 0.674, p < 0.001, 95% CI: 0.599–0.748, sensitivity = 72.7%, specificity = 46.9%) **(A)**. After POC treatment, 52 patients were included in the high QOL group and 248 patients in the low QOL group (AUC: 0.607, p = 0.010, 95% CI: 0.529–0.684, sensitivity = 37.5%, specificity = 15.6%) **(B)**.


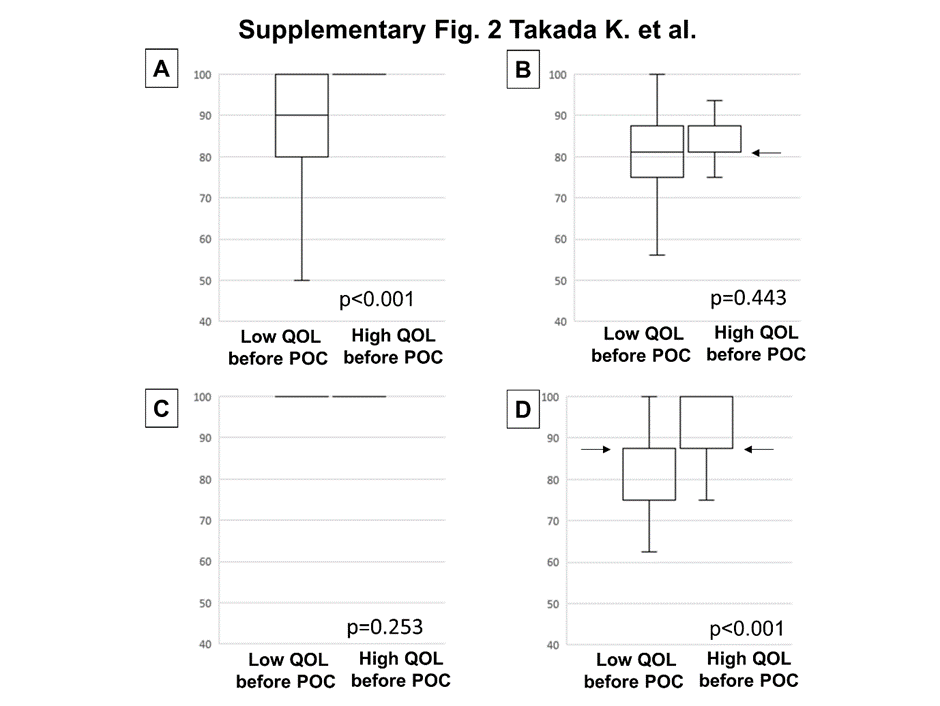


**Fig. S2** Comparison of high and low QOL groups on a subscale before POC. Before POC, when comparing high and low QOL groups on a subscale, the low QOL group was significantly lower in the “Physical symptoms and pain” (p < 0.01) **(A)**, and “Dress, sexual aspect, other” categories (p < 0.01) **(D)**, while “Satisfaction with treatment and coping with disease” (p = 0.44) **(B)** and “Side-effects of treatment” showed no change (p = 0.25) **(C)**. Where the box covers the bar for the median value, the position of the bar is indicated by an arrow


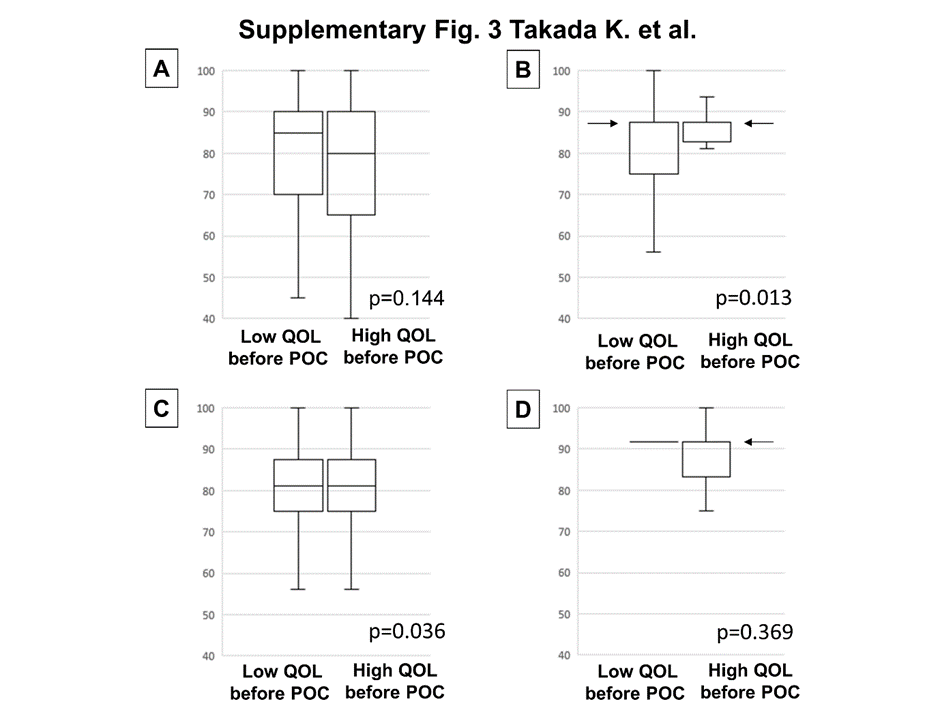


**Fig. S3** Comparison of subscales after POC in groups with high and low QOL before POC. After POC, when comparing high and low QOL groups before POC on a subscale, the low QOL group was significantly lower in the “Satisfaction with treatment and coping with disease” (p = 0.01) **(B)**, and “Side-effects of treatment” categories (p = 0.04) **(C)**, while “Physical symptoms and pain” (p = 0.11) **(A)** and “Dress, sexual aspect, other” showed no change (p = 0.37) **(D)**. **)**. Where the box covers the bar for the median value, the position of the bar is indicated by an arrow


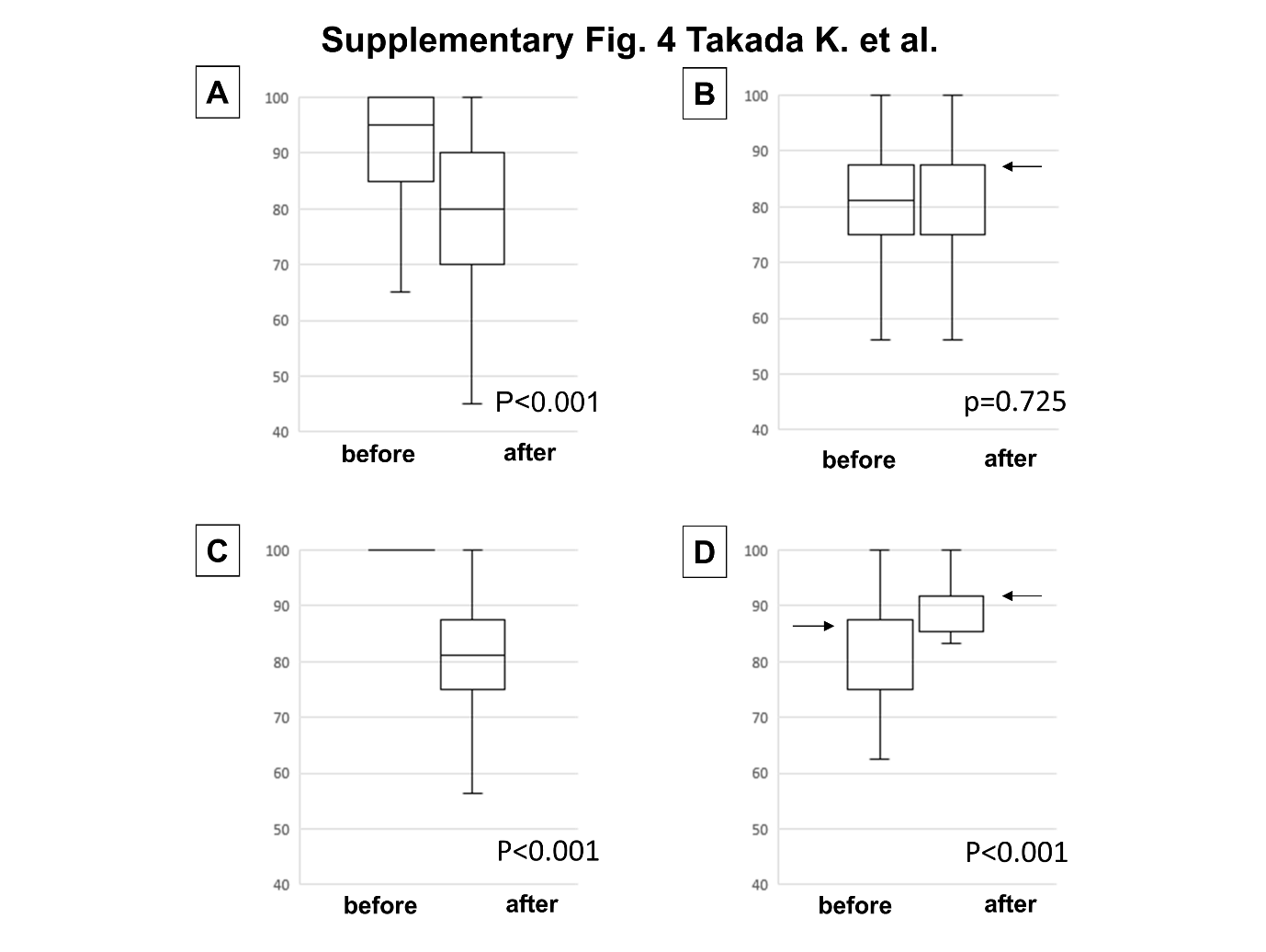


**Fig. S4** Changes in subscale QOL scores before and after POC. Each subscale QOL score before and after POC is showed by box-whisker plot diagram. There was a significant decrease in “Physical symptoms and pain” and “Side-effects of treatment” (p < 0.001) **(A)**, (p < 0.001) **(C)**, and “Satisfaction with treatment and coping with disease” showed no change (p = 0.725) **(B)**, while “Dress, sexual aspect, other” showed a significant increase (p < 0.001) **(D)**. **)**. Where the box covers the bar for the median value, the position of the bar is indicated by an arrow
